# Progression and Classification of Granular Osmiophilic Material (GOM) Deposits in Functionally Characterized Human *NOTCH3* Transgenic Mice

**DOI:** 10.1007/s12975-019-00742-7

**Published:** 2019-10-30

**Authors:** Gido Gravesteijn, Leon P. Munting, Maurice Overzier, Aat A. Mulder, Ingrid Hegeman, Marc Derieppe, Abraham J. Koster, Sjoerd G. van Duinen, Onno C. Meijer, Annemieke Aartsma-Rus, Louise van der Weerd, Carolina R. Jost, Arn M. J. M. van den Maagdenberg, Julie W. Rutten, Saskia A. J. Lesnik Oberstein

**Affiliations:** 1 Department of Clinical Genetics, Leiden University Medical Center, Albinusdreef 2, 2300 RC Leiden, The Netherlands; 2 Department of Radiology, Leiden University Medical Center, Albinusdreef 2, 2300 RC Leiden, The Netherlands; 3 Department of Human Genetics, Leiden University Medical Center, Albinusdreef 2, 2300 RC Leiden, The Netherlands; 4 Department of Cell and Chemical Biology, Leiden University Medical Center, Albinusdreef 2, 2300 RC Leiden, The Netherlands; 5 Department of Pathology, Leiden University Medical Center, Albinusdreef 2, 2300 RC Leiden, The Netherlands; 6 Department of Pediatric Neuro-Oncology, Prinses Máxima Center for Pediatric Oncology, Heidelberglaan 25, 3584 CS Utrecht, The Netherlands; 7 Department of Internal Medicine, Leiden University Medical Center, Albinusdreef 2, 2300 RC Leiden, The Netherlands; 8 Department of Neurology, Leiden University Medical Center, Albinusdreef 2, 2300 RC Leiden, The Netherlands

**Keywords:** CADASIL, Cerebral small vessel disease, Granular osmiophilic material (GOM), Electron microscopy (EM), NOTCH3, Cerebrovascular reactivity (CVR)

## Abstract

CADASIL is a *NOTCH3*-associated cerebral small vessel disease. A pathological ultrastructural disease hallmark is the presence of NOTCH3-protein containing deposits called granular osmiophilic material (GOM), in small arteries. How these GOM deposits develop over time and what their role is in disease progression is largely unknown. Here, we studied the progression of GOM deposits in humanized transgenic *NOTCH3*^Arg182Cys^ mice, compared them to GOM deposits in patient material, and determined whether GOM deposits in mice are associated with a functional CADASIL phenotype. We found that GOM deposits are not static, but rather progress in ageing mice, both in terms of size and aspect. We devised a GOM classification system, reflecting size, morphology and electron density. Six-month-old mice showed mostly early stage GOM, whereas older mice and patient vessels showed predominantly advanced stage GOM, but also early stage GOM. Mutant mice did not develop the most severe GOM stage seen in patient material. This absence of end-stage GOM in mice was associated with an overall lack of histological vascular pathology, which may explain why the mice did not reveal functional deficits in cerebral blood flow, cognition and motor function. Taken together, our data indicate that GOM progress over time, and that new GOM deposits are continuously being formed. The GOM staging system we introduce here allows for uniform GOM deposit classification in future mouse and human studies, which may lead to more insight into a potential association between GOM stage and CADASIL disease severity, and the role of GOM in disease progression.

**Electronic supplementary material:**

The online version of this article (10.1007/s12975-019-00742-7) contains supplementary material, which is available to authorized users.

## Introduction

Deposition of granular osmiophilic material (GOM) is the vascular pathological hallmark of CADASIL, which is the most prevalent hereditary small vessel disease [1] and is caused by missense mutations in the *NOTCH3* gene [2, 3]. GOM have been shown to contain NOTCH3 ectodomain (NOTCH3^ECD^) and extracellular matrix proteins [4–6], and can be visualized ultrastructurally in the tunica media of small arteries and capillaries. These electron dense GOM deposits are located in the basement membrane of mural cells, i.e. vascular smooth muscle cells and pericytes [7–11]. In both manifest and pre-manifest CADASIL patients, GOM deposits are present not only in brain vessels, but also in vessels of other organs, such as the skin [11–13]. Other CADASIL-associated vascular pathology includes mural cell degeneration, smooth muscle actin (SMA)-positive (neo)intima formation, fibrosis and vessel wall thickening [14–19]. These vascular alterations are associated with compromised cerebrovascular reactivity (CVR) [20, 21] and reduced cerebral blood flow (CBF), and eventually lead to mid-adult onset of recurrent strokes, vascular cognitive impairment and ultimately dementia [1]. Brain MRI reveals progressive symmetrical white matter hyperintensities, lacunes, microbleeds and brain atrophy [1].

We have previously described that our humanized CADASIL transgenic *NOTCH3*^Arg182Cys^ mouse model, which overexpresses human mutant NOTCH3 protein from a genomic construct, shows granular NOTCH3^ECD^ immunostaining as early as 4 weeks of age, while GOM deposits first appear around 6 months of age [22]. However, little is known about how GOM deposits evolve over time and what their relation is to other CADASIL-associated vascular pathology and vascular dysfunction. Here, we performed a longitudinal study of GOM pathology in the transgenic *NOTCH3*^Arg182Cys^ mice. In addition, we assessed cerebrovascular, motor and cognitive function in these mice.

## Methods

### Mice

Transgenic mice were used that harbour the human full-length *NOTCH3* gene (located on a 143 kb BAC construct) in either the wild-type or the mutant (c.544C>T, p.Arg182Cys) form, generated on a C57BL/6J background [22]. Mice were bred at the animal facility of the Leiden University Medical Center and housed individually under standard conditions, i.e. a 12-h light/dark cycle with food and water available ad libitum.

Three different mouse strains were used, with various human *NOTCH3* expression levels: 100% for wild-type mice (tgN3^WT^100), and 100% and 350% for mutant mice (tgN3^MUT^100 and tgN3^MUT^350, respectively) [22]. Non-transgenic littermates were used as additional controls. A prospective study with 6–8 mice per group was performed to study body weight and motor function at various time points (1.5, 3, 6, 12, 16 and 20 months), and cerebral hemodynamics, cognition and immunohistochemical staining was studied at 20 months. Three mice had to be sacrificed before the end of the study; one due to an eye infection (tgN3^WT^100, at 15 months), one due to having a wound on its back (tgN3^MUT^350, at 19 months) and one due to low body weight (tgN3^MUT^100, at 20 months). In addition to the prospective study, tgN3^MUT^350 mice were sacrificed at the age of 1.5 (*n* = 1), 3 (*n* = 1), 6 (*n* = 2), 12 (*n* = 2) and 20 (*n* = 2) months for electron microscopy (EM) studies.

### Electron Microscopy

In addition to mouse brain, post-mortem brain tissue was obtained from three CADASIL patients (deceased at age 59, 66 and 69 years) for comparison with human pathology. Mouse (frontal lobe grey matter) and human (frontal lobe grey matter) brain tissue was fixed overnight at 4 °C in 1.5% glutaraldehyde and 1% paraformaldehyde (pH = 7.4). Tissue blocks of ≤ 1 mm^3^ were post-fixated for 90 min in 2% osmium tetroxide and 2% potassium ferrocyanide after filtrating the post-fixative through a 0.2-μm filter. After post-fixation, the tissue was washed for 30 min in MilliQ and dehydrated in a series of ethanol (70%, 80% and 90%) for 30 min each and twice for 1 h in 100% ethanol. Blocks were incubated for 10 min in propylene oxide, 2 h in propylene oxide and Epon LX-112 (1:1) and finally for 2 h in propylene oxide and epon LX-112 (1:2). Subsequently, the epon was polymerized for 48 h at 70 °C.

One-micrometre-thick sections were checked for the presence of blood vessels by light microscopy. Then, areas with high blood vessel density were selected for further analysis with EM, i.e. 80-nm sections were collected on a one hole grid and subsequently stained with uranyl acetate and lead citrate. Images were acquired with a digital camera (One View, Gatan Inc., Pleasanton, CA) mounted on a 120 kV transmission electron microscope (Tecnai T12 with a twin objective lens, Fei Inc., Hillsburough, OR). Overviews of relatively large regions on the specimen that contained abundant numbers of cross-sections of vessels were collected by stitching many individual images together (40,000–60,000 nm^2^) using software described earlier [23]. Stitched images were examined using Aperio ImageScope (version 10.0.35).

### GOM Analysis

The number of GOM deposits was counted per vessel and expressed as counts per 100 μm vessel circumference. Vessel circumference was approximated using the formula of oval circumference (Ramanujan’s approximation for ellipse circumference = π[3(*a* + *b*) − √((3*a* + *b*) × (*a* + 3*b*))]) where *a* and *b* were defined as the major diameter (*a*) and minor diameter (*b*) for each vessel between endothelial basement membranes. GOM deposits were studied in vessels with a minor diameter < 8 μm, referred to as microvessels, as these were the most abundant in the stitched images. Twenty-three to 84 microvessels were studied per time point (1.5, 3, 6, 12 and 20 months). Width of the basement membrane was determined averaging 40 measurements in two ntg mice and two tgN3^MUT^350 mice each. In the human brain sample, 21 microvessels were analysed. Also, the GOM deposit area was measured using ImageJ after manually drawn region-of-interests around GOM deposits.

### Immunohistochemistry

Two 5-μm coronal frontal brain sections per mouse (approximately at the height of the infundibulum) and human brain sections of frontal white matter were analysed with immunohistochemistry. NOTCH3^ECD^ staining was performed as described before [22]. Smooth muscle actin (SMA) staining was performed after pre-treatment with trypsin for 30 min at 37 °C and washed three times for 5 min with PBS. The primary antibody (Alpha-Smooth Muscle Actin, 1:4000, goat polyclonal, NB300-978, Novus Biologicals) was incubated overnight at room temperature. The secondary antibody (Rabbit Anti-Goat IgG, biotinylated, 1:400, Jackson Immunoresearch Lab. Inc.) was incubated for 1 h at room temperature and developed with the Vectastain Elite ABC HRP Kit (PK-6100, Vectorlabs) for 30 min at room temperature. Finally, slices were stained with 0.05% 3,3′-diaminobenzidine (DAB, Sigma) supplemented with 0.0045% H_2_O_2_ for 10 min and stained with Harris’ haematoxylin solution (diluted 1:3, Merck) for 5 s. Sections were Verhoeff-Van Gieson and Periodic Schiff acid stained using the Artisan Link Pro staining machine (DAKO, Agilent), and Van Gieson stained as described previously [24]. Sections were stained with Klüver-Barrera luxol fast blue to quantify white matter vacuolization (Supplementary Methods 4).

Microscopy imaging was performed with the Keyence BZ-X710 (Keyence). Using 20 times magnification, full colour images were taken of the complete section at 1-ms capture time. Images were stitched by Keyence BZ-X Analyzer software version 1.3.0.3 to obtain one high resolution full brain image per section. The SMA positive area was determined using a Colour Threshold (Hue 0–50; Saturation 0–255; Brightness 0–175) in ImageJ, and expressed as percentage of total brain surface of the section.

### Neuroimaging, Cerebrovascular Reactivity, Cognition and Motor Function

At the age of 20 months, neuroimaging (T2W, FLAIR, SWI, cerebral hemodynamics) was performed under medetomidine anaesthesia using a 7 Tesla MRI (Bruker PharmaScan), see also Supplementary Methods 1. In short, absolute cerebral blood flow (CBF) was measured using arterial spin labelling (ASL)-MRI during a 7-min baseline and a 7-min CO_2_ challenge, using the measured signal difference between the labelled and control images in three brain slices. Absolute CBF at baseline and absolute CBF at challenge were quantified during the last 140 s of baseline and challenge, respectively. Absolute CBF increase was calculated as well as the cerebrovascular reactivity (CVR), which was defined as the relative cerebral blood flow (CBF) increase. One non-transgenic mouse was excluded from neuroimaging analysis due to a poor hemodynamic response to anaesthesia.

A Morris Water Maze protocol was used to assess cognitive function in 20-month-old mice, starting 2 weeks before neuroimaging assessment (Supplementary Methods 2). In short, during the training phase, mice were trained to find a hidden platform in the north-west quadrant of a circular swimming pool, while at the reversal training phase, mice were retrained to find a hidden platform in the south-east quadrant. Path length of the mice was determined between release in the pool and finding the hidden platform. Motor function was determined by analysing speed on a rotarod, on a beam and during swimming in the Morris water maze (Supplementary Methods 3). Motor and cognitive mouse experiments were performed by the same experienced researcher.

### Statistical Analyses

Differences between groups were analysed using one-way ANOVA analyses with Tukey’s post-hoc correction. The increase in GOM area over time, as well as the association between baseline CBF and CVR were analysed using simple linear regression. All statistical analyses were two-sided tests with threshold for statistical significance of 0.05, using the IBM SPSS Statistics version 23.0.0.2 software.

## Results

### Temporal GOM Assessment

**Fig. 1:**
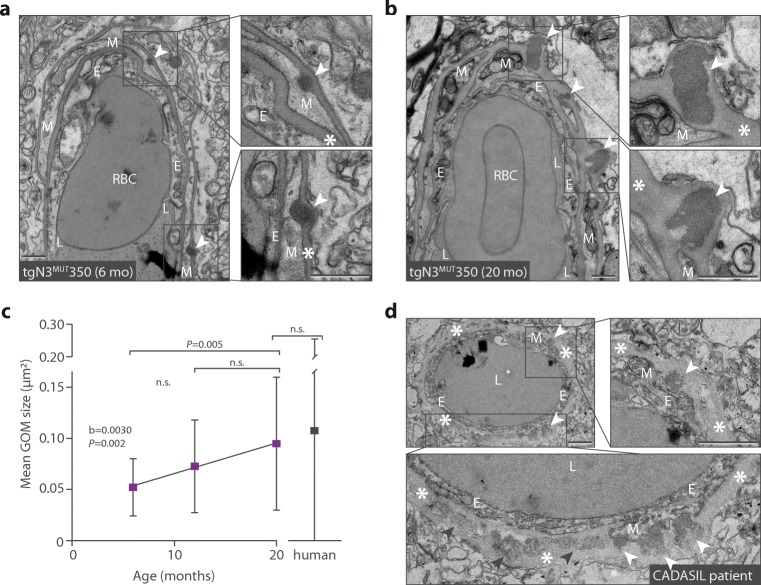
Progression of GOM in brain vessels of CADASIL mice. **a** Electron microscopy (EM) of brain vessels of tgN3^MUT^350 mice at the age of 6 months reveals round, electron dense GOM deposits near mural cells (white arrowheads). **b** EM at the age of 20 months shows larger and amorphous GOM deposits (white arrowheads), but also a small GOM deposit (grey arrowhead). The basement membrane bulges out at the location of a GOM deposit, but is otherwise not thickened in vessels of tgN3^MUT^350 mice. Mural cells have a normal aspect, i.e. without electron lucent vacuoles, large intracellular vesicles or a shrunken appearance. **c** Analysis of the size of GOM deposits in mouse brain vessels shows a significant increase in GOM size between the age of 6 and 20 months (*b* = 0.0030 μm^2^/month, *P* = 0.002). Average GOM size in brain vessels of deceased CADASIL patients (0.108 ± 0.148 μm^2^, *n* = 292 GOM deposits) was higher than in mice (0.095 ± 0.065 μm^2^, *n* = 31 GOM deposits), but this difference was not statistically significant (*P* = 0.634) due to the large variability in GOM size in human brain vessels. **d** Post-mortem analysis of CADASIL brain microvessels (*n* = 3 patients) showed extensive GOM pathology, with deposits in all vessels, both bulging into adjacent mural cells (white arrowheads) and located centrally in the thickened basement membrane further away from mural cells (grey arrowheads). In contrast to mice, GOM deposits in the CADASIL patient had a more granular aspect with heterogeneous electron density. CADASIL patient brain vessels had a thickened basement membrane and mural cells showed signs of degeneration, such as a shrunken appearance. *Arrowhead* GOM, *asterisk* basement membrane, *E* endothelial cell, *M* mural cell, *n*.*s*. non significant, *RBC* red blood cell. Bar represents 1 μm. Graph represents mean ± SD

**Fig. 2:**
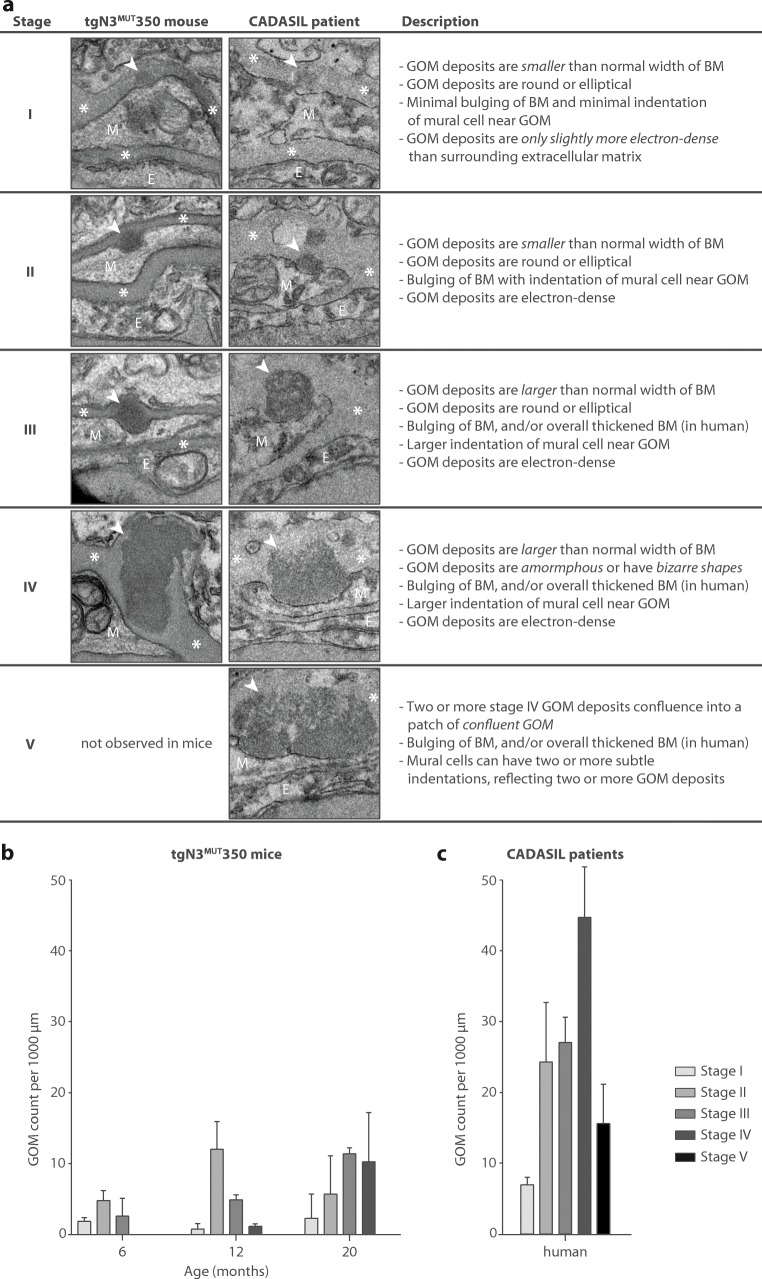
A five-stage GOM classification system for CADASIL. **a** Classification system for GOM deposits based on size, morphology and electron density. Per stage, examples are shown from brain vessels of tgN3^MUT^350 mice and of deceased CADASIL patient. In each example, the bottom of the image points towards the luminal side of the vessel. Cells were denoted as endothelial cells (E) or mural cells (M) based on interpretation of the morphology of cells as a whole within the vessel. **b** Staging of GOM deposits in brain vessels of tgN3^MUT^350 mice. At 6 months of age, stages I–III GOM deposits were present, while at 20 months mainly stages III–IV GOM deposits were observed. **c** Staging of GOM in brain vessels from deceased CADASIL patients. GOM deposits of all stages were observed, but stage IV GOM deposits were most abundant. Overall GOM count in patients was higher than in mice. *Arrowhead* GOM, *asterisk* basement membrane, *E* endothelial cell, *M* mural cell

GOM appearance, size and count were studied in brain microvessels of tgN3^MUT^350 mice at the age of 1.5, 3, 6, 12 and 20 months. GOM deposits were first observed at the age of 6 months, appearing as small, round deposits in the basement membrane of mural cells, which were sometimes only slightly more electron dense than the surrounding basement membrane (Fig. 1a). At 12 months, most GOM deposits were larger and more electron dense. At 20 months, large amorphous GOM deposits were observed, which spanned the full width of the basement membrane (Fig. 1b). Irrespective of age, GOM deposits were predominantly (85%) located on the abluminal side of mural cells, bulging out the basement membrane and thereby leaving an indentation in the adjacent mural cell. Between 6 and 20 months, GOM size increased (*b* = 0.0030 μm^2^/month, *P* = 0.002) (Fig. 1c), GOM count more than doubled (0.9 to 2.4 GOM/100 μm) and the percentage of GOM-positive vessels increased from 26 to 39%. Based on these observations, we categorized GOM deposits into various stages that reflect relative size, morphology, electron density and the GOM deposit-induced bulging of the basement membrane with concomitant indentation of adjacent mural cells (Fig. 2a). Stage I GOM deposits are small and slightly electron dense and induce minimal mural cell indentation. Stage II and III GOM deposits are more electron dense, induce basement membrane bulging with mural cell indentation and are within (II) or extend beyond (III) the normal width of the basement membrane (~ 150 nm). Stage IV GOM deposits extend beyond the normal width of the basement membrane and are amorphous. At 6 months of age, stage I–III GOM deposits were observed in tgN3^MUT^350 mice. At 12 months, the first stage IV GOM appeared. At 20 months, the majority of the GOM were at stages III and IV (Fig. 2b), but stage I GOM deposits were also observed. This suggests that new GOM deposits are continuously being formed, and that once formed, the initially small round GOM deposits progress over time into large amorphous deposits.

Next, we compared GOM in brain tissue of three CADASIL patients to GOM in 20-month-old tgN3^MUT^350 mice (Fig. 1d). Almost all (96%) of the analysed microvessels in the patients contained GOM deposits, whereas GOM deposits were observed in only 39% of microvessels in the mutant mice. In human brain material, like in mice, GOM deposits of all stages were observed, but stage IV GOM deposits were most frequent (Fig. 2c). In addition, the patients’ microvessels contained large confluent patches of GOM (stage V GOM) that were not observed in the mice. Of note, the electron density of GOM deposits in the patients’ microvessels was less homogeneous than of GOM in mice. GOM deposits in patient microvessels either bulged out of the basement membrane and thereby left an indentation in the adjacent mural cell, or were located further away from any recognisable mural cells within an overall thickened basement membrane. In contrast, GOM deposits in mice were always located close to the mural cell, often in indentations of the mural cell formed by the GOM deposits.

### Other CADASIL-Associated Vessel Wall and Brain Parenchyma Changes

**Fig. 3:**
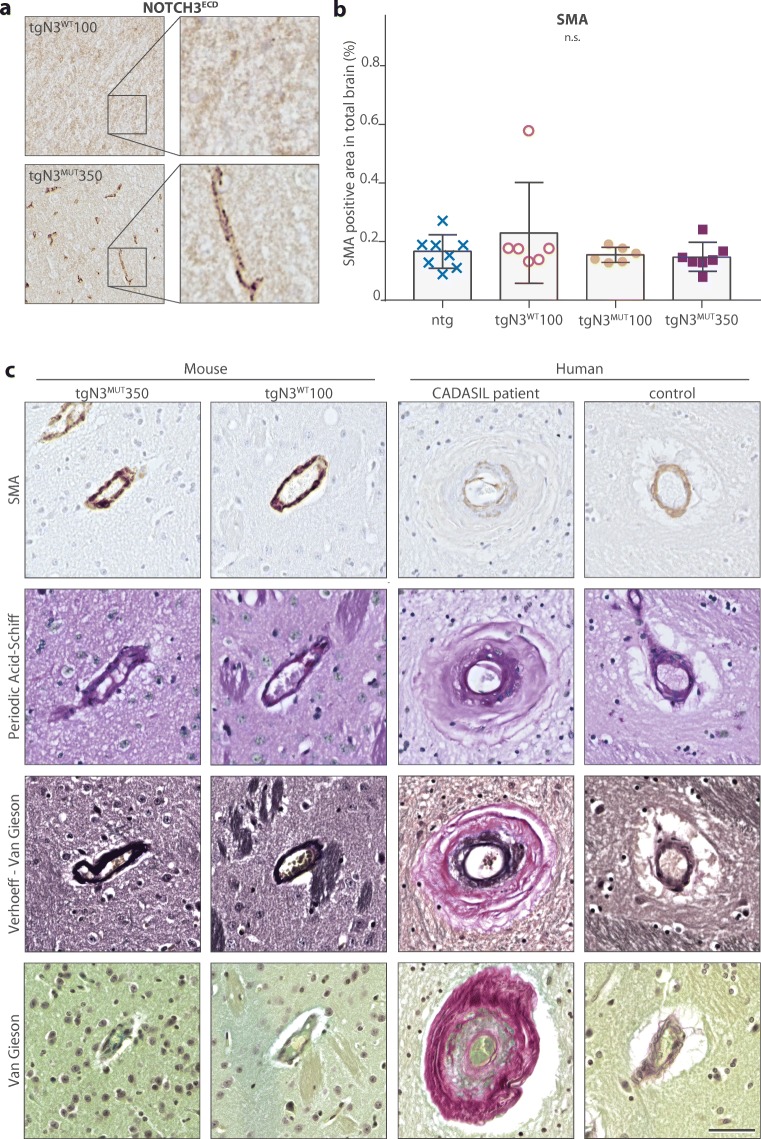
No vessel wall thickening or reduced SMA staining in mice expressing mutant human NOTCH3 protein. **a** Mutant mice (tgN3^MUT^350) show granular NOTCH3^ECD^ deposits in brain vessels, which were not observed in wild-type mice (tgN3^WT^100). **b** SMA content in brain was similar in the four mouse strains. One tgN3^WT^100 brain section contained a large artery which was longitudinally cleaved, resulting in a high SMA positive area. **c** The pattern of SMA immunoreactivity in the vessel wall was similar between the four groups (only images of the tgN3^MUT^350 and tgN3^WT^100 strains are shown), while the white matter of CADASIL patients showed less and fragmented SMA immunoreactivity, with SMA-positive areas in the intima. PAS, VvG and VG staining showed similar brain vessel staining patterns between the four groups, but the brain vessels of the CADASIL patients showed a clearly thickened vessel wall, with a positive PAS staining (carbohydrates), VvG staining (collagenous material red; elastic material black) and VG staining (collagenous material red). *SMA* smooth muscle actin, *n*.*s*. non significant, *PAS* periodic acid-Schiff staining, *VG* Van Gieson’s staining, *VVG* Verhoeff-Van Gieson’s staining. Bar represents 50 μm. Graph represents mean ± SD

Despite large GOM deposits and extensive granular NOTCH3^ECD^ staining at 20 months (Fig. 3a), tgN3^MUT^350 mice did not show basement membrane thickening (tgN3^MUT^350 0.145 ± 0.050 μm, ntg 0.148 ± 0.055 μm, *P* = 0.73) and there were no signs of mural degeneration, such as electron lucent vacuoles, large intracellular vesicles or a shrunken appearance (Fig. 1a, b). Also, there was no difference in the amount or pattern of SMA staining between mutant and wild-type mice (Fig. 3b, c). In addition, Van Gieson’s, Verhoeff-Van Gieson’s and Periodic acid-Schiff staining did not show vessel wall thickening, in contrast to our observations in vessels of the CADASIL patients (Fig. 3c). There was no difference in white matter vacuolization between wild-type and mutant mice (Supplementary Data 2).

### Cerebral Blood Flow, Cerebrovascular Reactivity and Neuroimaging

**Fig. 4:**
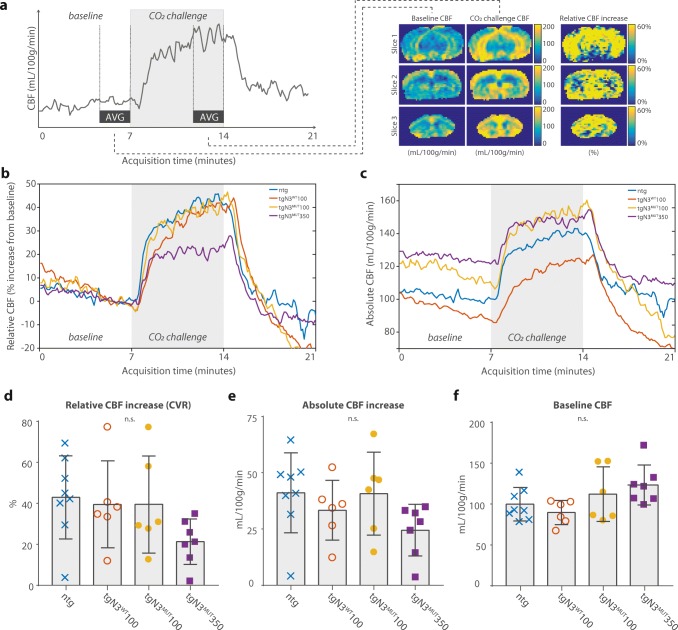
CADASIL mice do not show altered cerebral hemodynamics. **a** Representative CBF profile over time, and three slices of CBF images at baseline, at CO_2_-challenge and relative CBF increase (CVR) are shown. **b** Average relative CBF profiles in tgN3^WT^100, tgN3^MUT^100, tgN3^MUT^350 and ntg mice. **c** Average absolute CBF profiles in tgN3^WT^100, tgN3^MUT^100, tgN3^MUT^350 and ntg mice. **d** Relative CBF increase (CVR) was similar in the four groups (ntg: 43.0 ± 20.3%; tgN3^WT^100: 39.5 ± 21.2%; tgN3^MUT^100: 39.5 ± 23.7%; tgN3^MUT^350: 21.3 ± 11.1%; *P* = 0.17; ANOVA), although a non-significant trend of lower CVR in the tgN3^MUT^350 mice compared to ntg was observed. **e** Absolute CBF rise upon CO_2_ challenge was similar in the four groups (ntg: 41.1 ± 17.8 mL/100 g/min; tgN3^WT^100: 33.4 ± 13.3 mL/100 g/min; tgN3^MUT^100: 40.7 ± 18.5 mL/100 g/min; tgN3^MUT^350: 24.5 ± 11.5 mL/100 g/min; *P* = 0.18; ANOVA]). **f** Absolute baseline CBF was similar in the four groups, although tgN3^MUT^350 showed a non-significant higher baseline CBF (ntg: 99.9 ± 20.5 mL/100 g/min; tgN3^WT^100: 89.7 ± 14.7 mL/100 g/min; tgN3^MUT^100: 112.2 ± 33.5 mL/100 g/min; tgN3^MUT^350: 123.5 ± 24.4 mL/100 g/min; *P* = 0.09; ANOVA). Physiology parameters did not differ between groups (Supplementary Data 7 and 8). Body weight was different between groups (Supplementary Data 9), but CBF and CVR analyses corrected for weight gave similar results. *AVG* average, *CBF* cerebral blood flow, *CVR* cerebrovascular reactivity, *n*.*s*. non significant. Graphs represent mean or mean ± SD

Next, we assessed cerebral blood flow (CBF) dynamics in 20-month-old mice using ASL-MRI with CO_2_ as vasodilative stimulus (Fig. 4a). Compared to non-transgenic and wild-type mice, mutant mice (tgN3^MUT^100 and tgN3^MUT^350) did not show a significant difference in CVR, which was defined as relative CBF increase upon challenge (Fig. 4b, d), absolute CBF increase upon challenge (Fig. 4c, e) or in CBF at baseline (Fig. 4f). There was, however, a non-significant trend towards a reduced CVR and a slightly increased baseline CBF in the tgN3^MUT^350 mice. Differences in baseline CBF likely contributed to the observed differences in CVR, as there was an inverse correlation between baseline CBF and CVR (s = − 0.69, *P* < 0.001, Supplementary data 1). Separate analysis of CBF and CVR for cortex and subcortex showed similar results (Supplementary data 3, 4).

High-resolution T2W, FLAIR and SWI MRI scans of mouse brains did not show white matter hyperintensities, lacunes or microbleeds. Also, gadolinium enhancement showed the typical pattern of contrast enhancement in and around the ventricles, but no differences were observed between tgN3^MUT^350 and wild-type mice (Supplementary data 5).

### Cognition and Motor Function

**Fig. 5:**
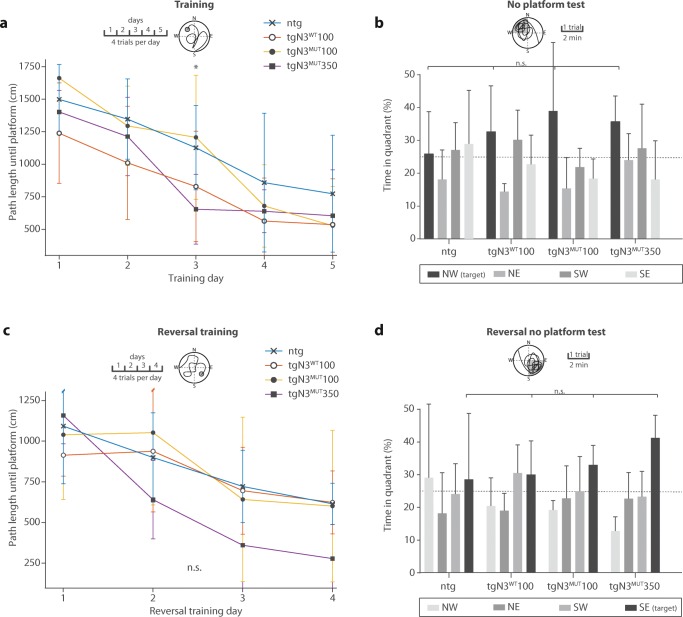
CADASIL mice do not show cognitive dysfunction. **a** The path length until finding a hidden platform in the water maze was similar for all strains (ntg, tgN3^WT^100, tgN3^MUT^100, tgN3^MUT^350). **b** After the five training days, mice underwent one trial without a platform. The time spent in the target quadrant (NW) was similar between the groups (*P* = 0.46; ANOVA). **c** A 4-day reversal training phase with the platform hidden in the south-east quadrant showed no significant differences in path length until finding the hidden platform between the groups (*P* = 0.14 on day 4; ANOVA). **d** Time in target quadrant after the reversal training was similar between the groups (*P* = 0.09; ANOVA). Graphs represent mean ± SD; *n*.*s*. non significant; *P < 0.05

Motor function, as assessed by rotarod running, beam walk and swimming speed, did not differ between mutant and wild-type mice at any of the time points (Supplementary data 6). In the Morris water maze tests, mutant mice did not show signs of impaired memory formation. If anything, tgN3^MUT^350 mice performed slightly better at some of the tasks (Fig. 5).

## Discussion

We investigated the development of GOM deposits over time in a transgenic mouse model of CADASIL, which overexpresses mutant human NOTCH3 protein from a large genomic construct. GOM deposits evolved with respect to size, morphology and number in microvessels of the mutant mouse brain. Here, we propose a five-stage GOM classification system to facilitate uniform analysis and description of GOM deposits and show that this staging can also be used to systematically classify GOM in vessels of CADASIL patient material.

The GOM deposits observed in aged mice (20 months) included stages I–IV, i.e. from small, circumscript deposits within the basement membrane (stage I), to large, amorphous GOM that induced bulging of the basement membrane (stage IV). As mice aged 6 months only showed stages I–III GOM, individual GOM deposits seem to increase in size and become increasingly amorphous over time, and new GOM seem to be continuously formed. This is further illustrated by the observation that GOM deposits of stage I and II are also present in post-mortem CADASIL patient brain microvessels, next to the more extensive GOM pathology, including patches of confluent GOM (stage V) [7, 25, 26].

Although many studies have reported the presence and morphology of GOM in CADASIL patients [9, 11–13, 25, 27–30], little is known about how GOM deposits progress over time. Brulin et al. analysed GOM in skin biopsies of CADASIL patients of different ages, and found that the number GOM deposits increase up to 50 years of age, but also found that the number of GOM seems to decrease in elderly patients [11]. The latter may either be attributed to other end-stage vessel wall changes hampering the visualization of GOM, or because GOM seem to become confluent and disintegrate over time [26]. Whether different GOM stages in skin biopsies of CADASIL patients are associated with disease severity and disease progression, therefore, remains to be determined. If so, the proposed GOM classification system may aid future efforts to monitor and predict disease progression at the individual patient level.

In our humanized CADASIL mouse model and in a rat Notch3 CADASIL mouse model, GOM deposits are observed several months after the first signs of NOTCH3^ECD^-positive granular immunostaining [22, 30], suggesting that NOTCH3^ECD^ granules may act as seeds for GOM development. Since NOTCH3^ECD^ aggregates attract extracellular matrix proteins that are components of GOM deposits, such as TIMP3 and clusterin [4, 5], there seems a direct temporal relation between NOTCH3^ECD^ aggregates and the formation of GOM deposits. Delineating molecular differences between early- and end-stage GOM deposits may help to understand the sequence of events in CADASIL vascular pathology, and perhaps the identification of early therapeutic targets.

Whereas the CADASIL mice in our study seem to faithfully replicate early signs of disease pathology (NOTCH3^ECD^ accumulation and GOM deposition), we did not observe other CADASIL-associated disease features which have been observed in other CADASIL mouse models, such as vessel wall thickening, changes in SMA staining, mural cell degeneration and blood-brain barrier leakage [7, 9, 14, 15, 17, 31]. In line with this, we previously showed that our mutant mice do not show brain parenchyma pathology at age 20 months, which we confirmed in this study using high-resolution T2W neuroimaging [22]. Furthermore, we extended the characterization of the mice with functional tests, which did not reveal any cognitive or motor dysfunction. Our cerebral blood flow studies showed a small, not significant, reduction in CVR in tgN3^MUT^350 mice, while studies in other, genetically different, CADASIL mouse models did show a reduced CVR [8, 30, 32]. It may be relevant that we used a different method to anaesthetize mice and also to measure CVR, namely an ASL-based MRI approach. Although less sensitive in detecting small CVR changes, the advantage of ASL-MRI is that it allows for absolute perfusion quantification and detection of differences in baseline CBF. In that way, we found that the slight reduction of CVR in tgN3^MUT^350 mice could at least partially be explained by a higher CBF at baseline. Future research is needed to determine whether the differences in CVR findings between this study and others [8, 30, 32] can be explained by differences in genetics of the mouse models, by differences in baseline perfusion states or by differences in the study set-up, including the anaesthesia protocol. Although we did not find evidence for overt functional cerebrovascular deficits in our mouse model, this humanized mouse model captures early markers of CADASIL pathology, making it suitable for therapeutic studies targeting human mutant NOTCH3^ECD^ accumulation early in the disease course.

In summary, we show progression of GOM deposits in a humanized CADASIL mouse model. We propose a five-stage GOM classification system for uniform assessment of GOM depositions in translational research. In future pre-clinical studies of therapeutic approaches aimed at reducing or preventing NOTCH3^ECD^ aggregation, GOM classification may serve as a valuable tool to monitor therapeutic efficiency on an ultrastructural level.

## Electronic Supplementary Material

ESM 1 (DOCX 2128 kb)

ESM 2 (DOCX 94 kb)

## References

[1] Chabriat H, Joutel A, Dichgans M, Tournier-Lasserve E, Bousser M-G (2009). Cadasil. Lancet Neurol.

[2] Joutel A, Vahedi K, Corpechot C, Troesch A, Chabriat H, Vayssière C (1997). Strong clustering and stereotyped nature of Notch3 mutations in CADASIL patients. Lancet..

[3] Joutel A, Corpechot C, Ducros A, Vahedi K, Chabriat H, Mouton P (1996). Notch3 mutations in CADASIL, a hereditary adult-onset condition causing stroke and dementia. Nature..

[4] Ishiko A, Shimizu A, Nagata E, Takahashi K, Tabira T, Suzuki N (2006). Notch3 ectodomain is a major component of granular osmiophilic material (GOM) in CADASIL. Acta Neuropathol.

[5] Monet-Leprêtre M, Haddad I, Baron-Menguy C, Fouillot-Panchal M, Riani M, Domenga-Denier V (2013). Abnormal recruitment of extracellular matrix proteins by excess Notch3 ECD: a new pathomechanism in CADASIL. Brain..

[6] Arboleda-Velasquez JF, Manent J, Lee JH, Tikka S, Ospina C, Vanderburg CR (2011). Hypomorphic notch 3 alleles link notch signaling to ischemic cerebral small-vessel disease. Proc Natl Acad Sci.

[7] Yamamoto Y, Craggs LJL, Watanabe A, Booth T, Attems J, Low RWC (2013). Brain microvascular accumulation and distribution of the NOTCH3 ectodomain and granular osmiophilic material in CADASIL. J Neuropathol Exp Neurol.

[8] Ghosh M, Balbi M, Hellal F, Dichgans M, Lindauer U, Plesnila N (2015). Pericytes are involved in the pathogenesis of cerebral autosomal dominant arteriopathy with subcortical infarcts and leukoencephalopathy. Ann Neurol.

[9] Gu X, Liu X-Y, Fagan A, Gonzalez-Toledo ME, Zhao L-R (2012). Ultrastructural changes in cerebral capillary pericytes in aged Notch3 mutant transgenic mice. Ultrastruct Pathol.

[10] Joutel A, Andreux F, Gaulis S, Domenga V, Cecillon M, Battail N (2000). The ectodomain of the Notch3 receptor accumulates within the cerebrovasculature of CADASIL patients. J Clin Invest.

[11] Brulin P, Godfraind C, Leteurtre E, Ruchoux M-M (2002). Morphometric analysis of ultrastructural vascular changes in CADASIL: analysis of 50 skin biopsy specimens and pathogenic implications. Acta Neuropathol.

[12] Tikka S, Mykknen K, Ruchoux MM, Bergholm R, Junna M, Pyhnen M (2009). Congruence between NOTCH3 mutations and GOM in 131 CADASIL patients. Brain..

[13] Morroni M, Marzioni D, Ragno M, Di Bella P, Cartechini E, Pianese L (2013). Role of Electron microscopy in the diagnosis of Cadasil syndrome: a study of 32 patients. PLoS One.

[14] Ruchoux MM, Guerouaou D, Vandenhaute B, Pruvo J-PP, Vermersch P, Leys D (1995). Systemic vascular smooth muscle cell impairment in cerebral autosomal dominant arteriopathy with subcortical infarcts and leukoencephalopathy. Acta Neuropathol.

[15] Kalimo H, Viitanen M, Amberla K, Juvonen V, Marttila R, Pöyhönen M (1999). CADASIL: hereditary disease of arteries causing brain infarcts and dementia. Neuropathol Appl Neurobiol.

[16] Miao Q, Paloneva T, Tuisku S, Roine S, Poyhonen M, Viitanen M (2006). Arterioles of the lenticular nucleus in CADASIL. Stroke..

[17] Gatti JR, Zhang X, Korcari E, Lee SJ, Greenstone N, Dean JG (2019). Redistribution of mature smooth muscle markers in brain arteries in cerebral autosomal dominant arteriopathy with subcortical infarcts and Leukoencephalopathy. Transl Stroke Res.

[18] Yamamoto Y, Ihara M, Tham C, Low RWC, Slade JY, Moss T (2009). Neuropathological correlates of temporal pole white matter hyperintensities in CADASIL. Stroke..

[19] Dong H, Ding H, Young K, Blaivas M, Christensen PJ, Wang MM (2013). Advanced intimal hyperplasia without luminal narrowing of leptomeningeal arteries in CADASIL. Stroke..

[20] Pfefferkorn T, von Stuckrad-Barre S, Herzog J, Gasser T, Hamann GF, Dichgans M (2001). Reduced cerebrovascular CO(2) reactivity in CADASIL: a transcranial Doppler sonography study. Stroke..

[21] Chabriat H, Pappata S, Ostergaard L, Clark CA, Pachot-Clouard M, Vahedi K (2000). Cerebral hemodynamics in CADASIL before and after acetazolamide challenge assessed with MRI bolus tracking. Stroke..

[22] Rutten JW, Klever RR, Hegeman IM, Poole DS, Dauwerse HG, LAM B (2015). The NOTCH3 score: a pre-clinical CADASIL biomarker in a novel human genomic NOTCH3 transgenic mouse model with early progressive vascular NOTCH3 accumulation. Acta Neuropathol Commun.

[23] Faas FGA, Cristina Avramut M, van den Berg BM, Mieke Mommaas A, Koster AJ, Ravelli RBG (2012). Virtual nanoscopy: generation of ultra-large high resolution electron microscopy maps. J Cell Biol.

[24] Dodds HM, Clark G (1985). An improved Van Gieson. Stain Technol.

[25] Lewandowska E, Dziewulska D, Parys M, Pasennik E (2011). Ultrastructure of granular osmiophilic material deposits (GOM) in arterioles of CADASIL patients. Folia Neuropathol.

[26] Lewandowska E, Felczak P, Buczek J, Gramza K, Rafałowska J (2014). Blood vessel ultrastructural picture in a CADASIL patient diagnosed at an advanced age. Folia Neuropathol.

[27] Ruchoux MM, Domenga V, Brulin P, Maciazek J, Limol S, Tournier-Lasserve E (2003). Transgenic mice expressing mutant Notch3 develop vascular alterations characteristic of cerebral autosomal dominant arteriopathy with subcortical infarcts and leukoencephalopathy. Am J Pathol.

[28] Monet M, Domenga V, Lemaire B, Souilhol C, Langa F, Babinet C (2007). The archetypal R90C CADASIL-NOTCH3 mutation retains NOTCH3 function in vivo. Hum Mol Genet.

[29] Monet-Lepretre M, Bardot B, Lemaire B, Domenga V, Godin O, Dichgans M (2009). Distinct phenotypic and functional features of CADASIL mutations in the Notch3 ligand binding domain. Brain..

[30] Joutel A, Monet-Leprêtre M, Gosele C, Baron-menguy C, Hammes A, Schmidt S (2010). Cerebrovascular dysfunction and microcirculation rarefaction precede white matter lesions in a mouse genetic model of cerebral ischemic small vessel disease. J Clin Invest.

[31] Miao Q, Paloneva T, Tuominen S, Pöyhönen M, Tuisku S, Viitanen M (2006). Fibrosis and stenosis of the long penetrating cerebral arteries: the cause of the white matter pathology in cerebral autosomal dominant arteriopathy with subcortical infarcts and leukoencephalopathy. Brain Pathol.

[32] Lacombe P, Oligo C, Domenga V, Tournier-Lasserve E, Joutel A (2005). Impaired cerebral vasoreactivity in a transgenic mouse model of cerebral autosomal dominant arteriopathy with subcortical infarcts and leukoencephalopathy arteriopathy. Stroke..

