# Supplementary material for: Progression and Classification of Granular Osmiophilic Material (GOM) Deposits in Functionally Characterized Human NOTCH3 Transgenic Mice
Source: Transl Stroke Res. 2019 Oct 30;11(3):517–27. doi: 10.1007/s12975-019-00742-7 (PMC7235067; doi:10.1007/s12975-019-00742-7)
Supplement: Supplementary file 1 — (DOCX 2128 kb) [file 12975_2019_742_MOESM1_ESM.docx]

# Supplementary Data

**Progression and classification of granular osmiophilic material (GOM) deposits in functionally characterized human NOTCH3 transgenic mice**

*Running head: GOM stages in CADASIL*

*Translational Stroke Research*

Gido Gravesteijn^1^, Leon P. Munting^2^, Maurice Overzier^3^, Aat A. Mulder^4^, Ingrid Hegeman^5^, Marc Derieppe^2,6^, Abraham J. Koster^4^, Sjoerd G. van Duinen^5^, Onno C. Meijer^7^, Annemieke Aartsma-Rus^3^, Louise van der Weerd^2,3^, Carolina R. Jost^4^_,_ Arn M.J.M. van den Maagdenberg^3,8^, Julie W. Rutten^1,3*^, Saskia A.J. Lesnik Oberstein^1*§^

^1^ Department of Clinical Genetics, Leiden University Medical Center, Albinusdreef 2, 2300 RC Leiden, The Netherlands.
^2^ Department of Radiology, Leiden University Medical Center, Albinusdreef 2, 2300 RC Leiden, The Netherlands.
^3^ Department of Human Genetics, Leiden University Medical Center, Albinusdreef 2, 2300 RC Leiden, The Netherlands.
^4^ Department of Cell and Chemical Biology, Leiden University Medical Center, Albinusdreef 2, 2300 RC Leiden, The Netherlands.
^5^ Department of Pathology, Leiden University Medical Center, Albinusdreef 2, 2300 RC Leiden, The Netherlands.
^6^ Department of Pediatric Neuro-Oncology, Prinses Máxima Center for Pediatric Oncology, Heidelberglaan 25, 3584 CS, Utrecht, The Netherlands.
^7^ Department of Internal Medicine, Leiden University Medical Center, Albinusdreef 2, 2300 RC Leiden, The Netherlands.
^8^ Department of Neurology, Leiden University Medical Center, Albinusdreef 2, 2300 RC Leiden, The Netherlands.
* Shared last authorship.
^§^ Corresponding author: Saskia Lesnik Oberstein, [lesnik@lumc.nl](mailto:lesnik@lumc.nl).

## Content

Suppl. Data 1: Baseline CBF correlates with CVR in CADASIL mice p.2
Suppl. Data 2: CADASIL mice do not show increased levels of vacuolization p.3
Suppl. Data 3: CADASIL mice do not show altered cortical cerebrovascular reactivity p.4
Suppl. Data 4: CADASIL mice do not show altered subcortical cerebrovascular reactivity p.5
Suppl. Data 5: CADASIL mice do not show blood brain barrier leakage p.6
Suppl. Data 6: CADASIL mice do not show motor dysfunction p.7
Suppl. Data 7: Physiology parameters during cerebral hemodynamic assessment
 at baseline and at CO_2_ challenge in CADASIL mice p.8
Suppl. Data 8: pCO2 in blood increase upon CO¬2 challenge p.9
Suppl. Data 9: Reduced body weight in tgN3^MUT^350 and tgN3^WT^100 mice
 compared to ntg and tgN3^MUT^100 mice p.10

**
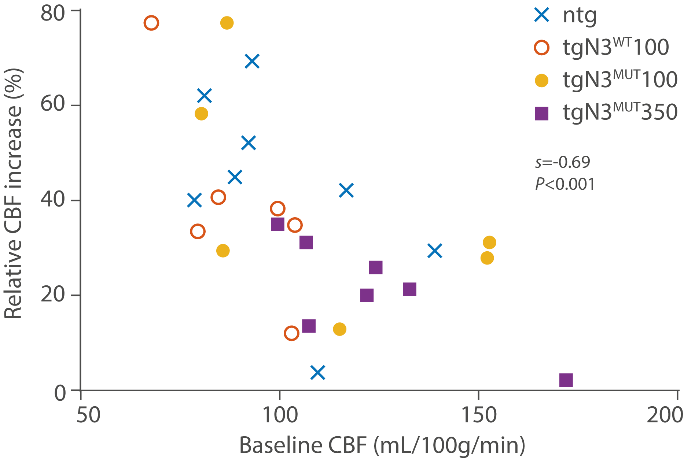
**

**Supplementary Data 1: Baseline CBF correlates with CVR in CADASIL mice**

Relative CBF increase (CVR) was determined for all mouse strains (ntg, tgN3^WT^100, tgN3^MUT^100, tgN3^MUT^350). CVR values were negatively associated with baseline CBF values (s=-0.69, *P*<0.001), suggesting that CVR represents differences in both baseline CBF and actual vascular reactivity.

**
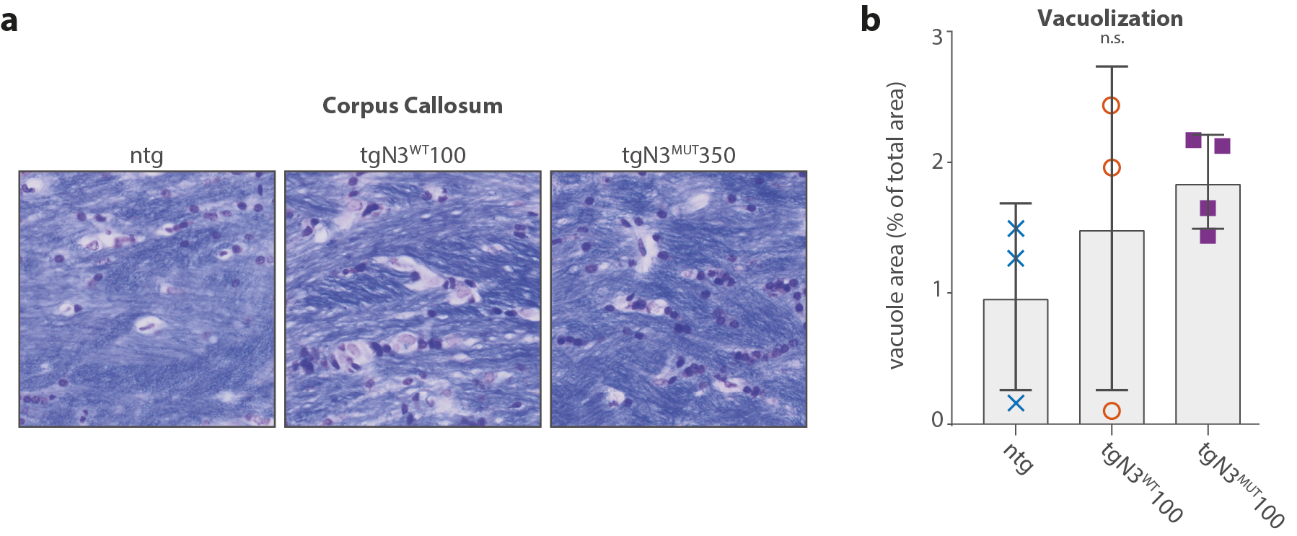
**

**Supplementary Data 2: CADASIL mice do not show increased levels of vacuolization**

(A) Representative images of Klüver-Barrera luxol fast blue staining of the corpus callosum in 20-month-old non-transgenic mice (ntg), wildtype mice (tgN3^WT^100) and mutant mice (tgN3^MUT^350). (B) Quantification of the vacuole area in the corpus callosum, showing similar levels of vacuolization (ntg 0.98%±0.71%, tgN3^WT^100 1.51%±0.71%, tgN3^MUT^350 1.86%±0.36%, *P*=0.40, ANOVA). Graph represent mean±SD; n.s. = non significant.

**
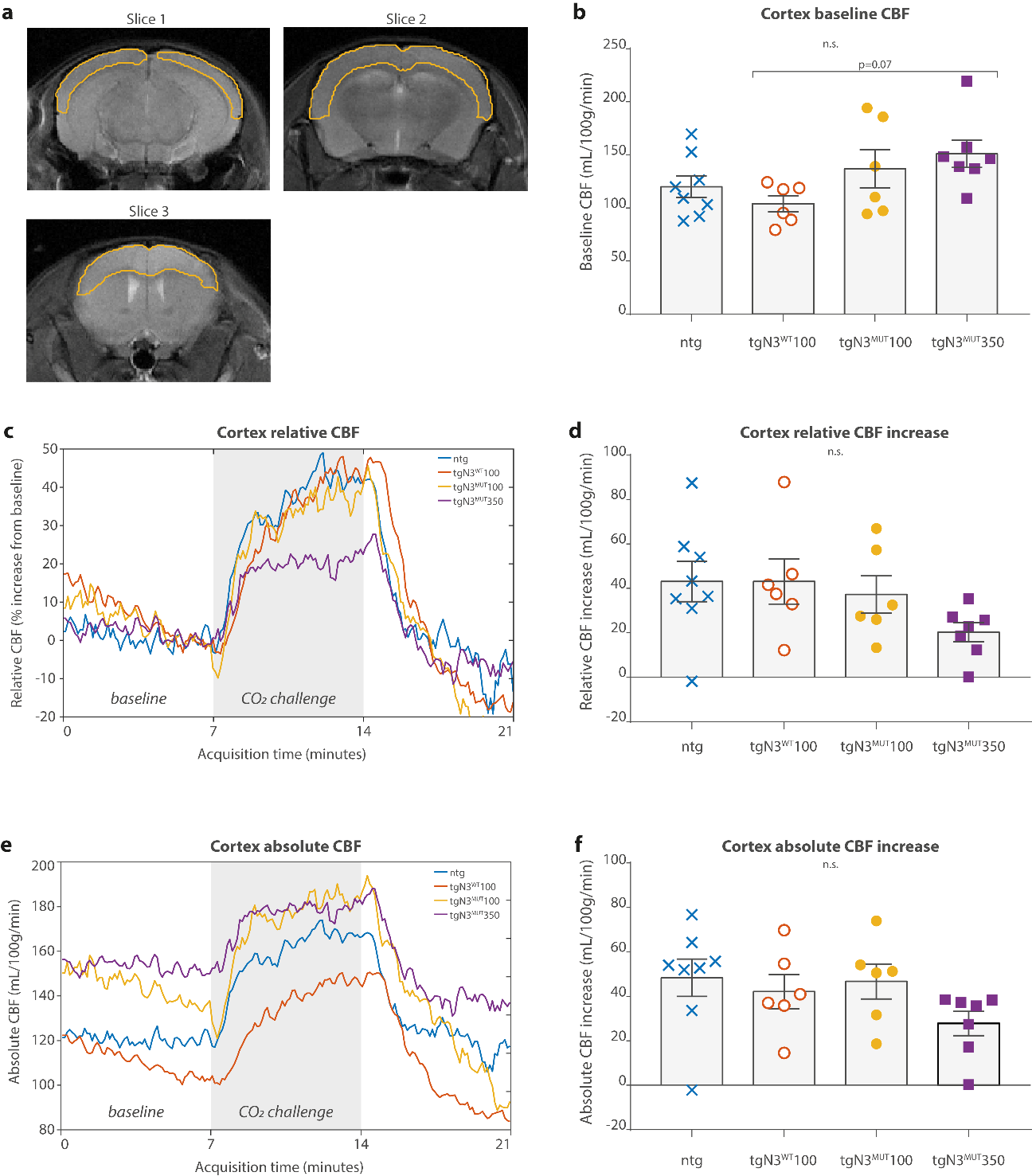
**

**Supplementary Data 3: CADASIL mice do not show altered cortical cerebrovascular reactivity**

**(A)** Region-of-interest of cortex. **(B)** Baseline CBF in cortex was similar among the groups (ntg, tgN3^WT^100, tgN3^MUT^100, tgN3^MUT^350). **(C,D)** Average relative CBF profiles over time are shown for cortex. CVR was similar in all groups. **(E,F)** Average absolute CBF profiles over time. Absolute CBF rise upon CO_2_ challenge was similar between the groups. Graphs represent mean or mean±SD; n.s. = non significant.

**
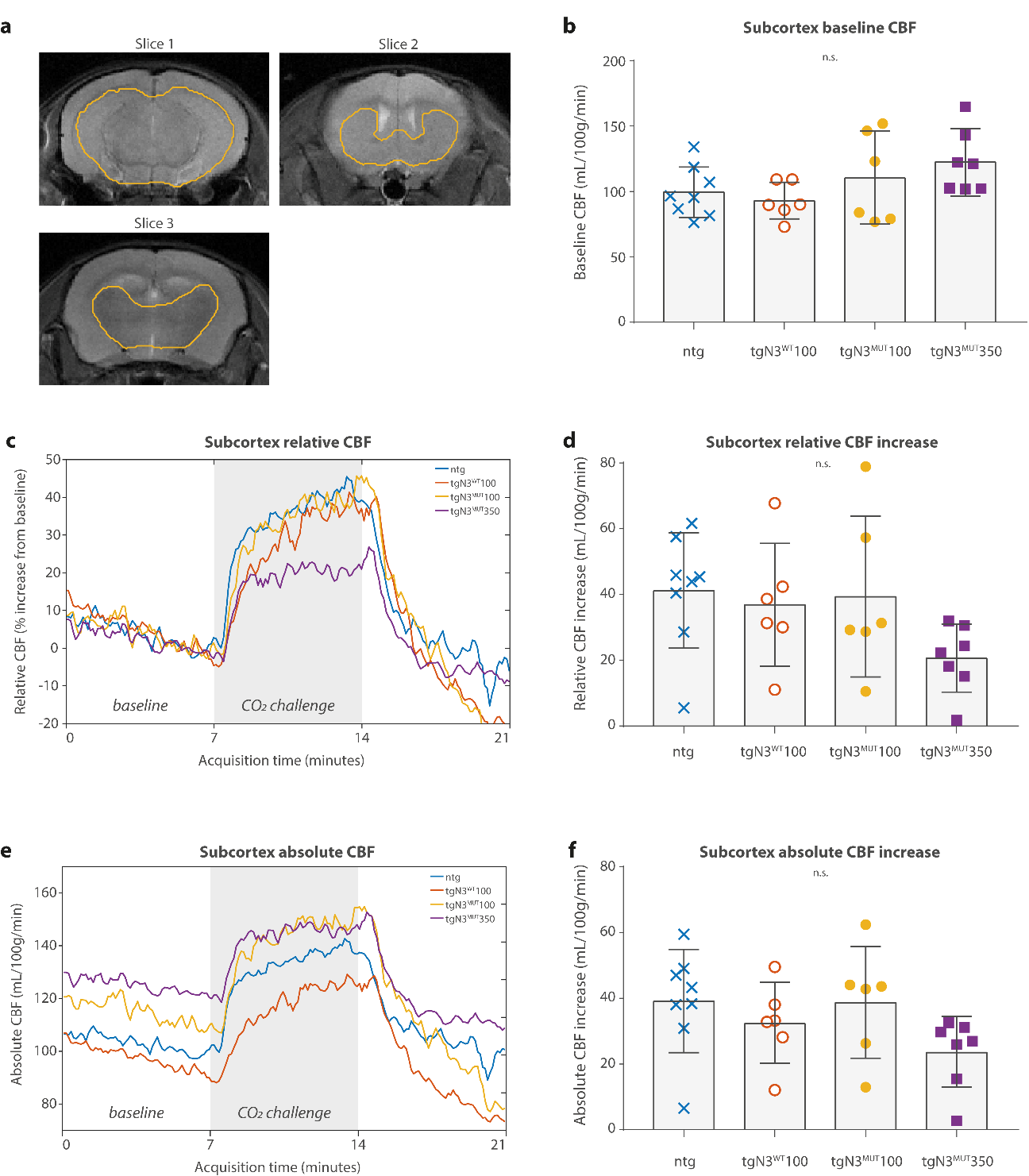
**

**Supplementary Data 4: CADASIL mice do not show altered subcortical cerebrovascular reactivity**

**(A)** Region-of-interest of subcortex. **(B)** Baseline CBF in subcortex was similar among the groups (ntg, tgN3^WT^100, tgN3^MUT^100, tgN3^MUT^350). **(C,D)** Average relative CBF profiles over time are shown for subcortex. CVR was similar in all groups. **(E,F)** Average absolute CBF profiles over time. Absolute CBF rise upon CO_2_ challenge was similar between the groups. Graphs represent mean or mean±SD; n.s. = non significant.

**
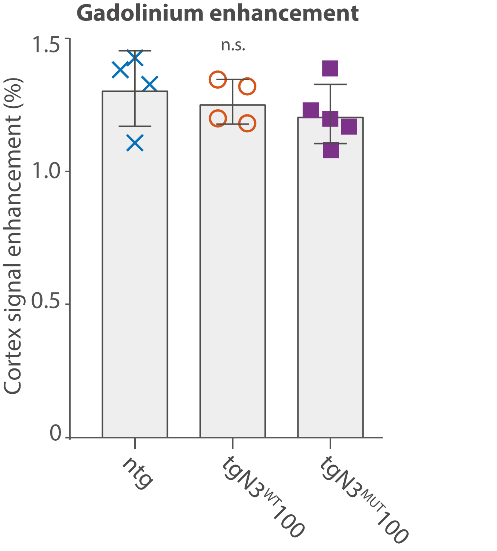
**

**Supplementary Data 5: CADASIL mice do not show blood brain barrier leakage**

Blood brain barrier function was assessed in a second cohort of ntg (n=4), tgN3^WT^100 (n=4), and tgN3^MUT^350 (n=5) mice at the age of 12 months by determining Gadolinium-induced signal enhancement on brain MRI after injection of Gadolinium. No differences in signal enhancement were observed (ntg 1.31%±0.14%, tgN3^WT^100 1.26%±0.08%, tgN3^MUT^350 1.22%±0.11%, *P*=0.48, ANOVA).

**
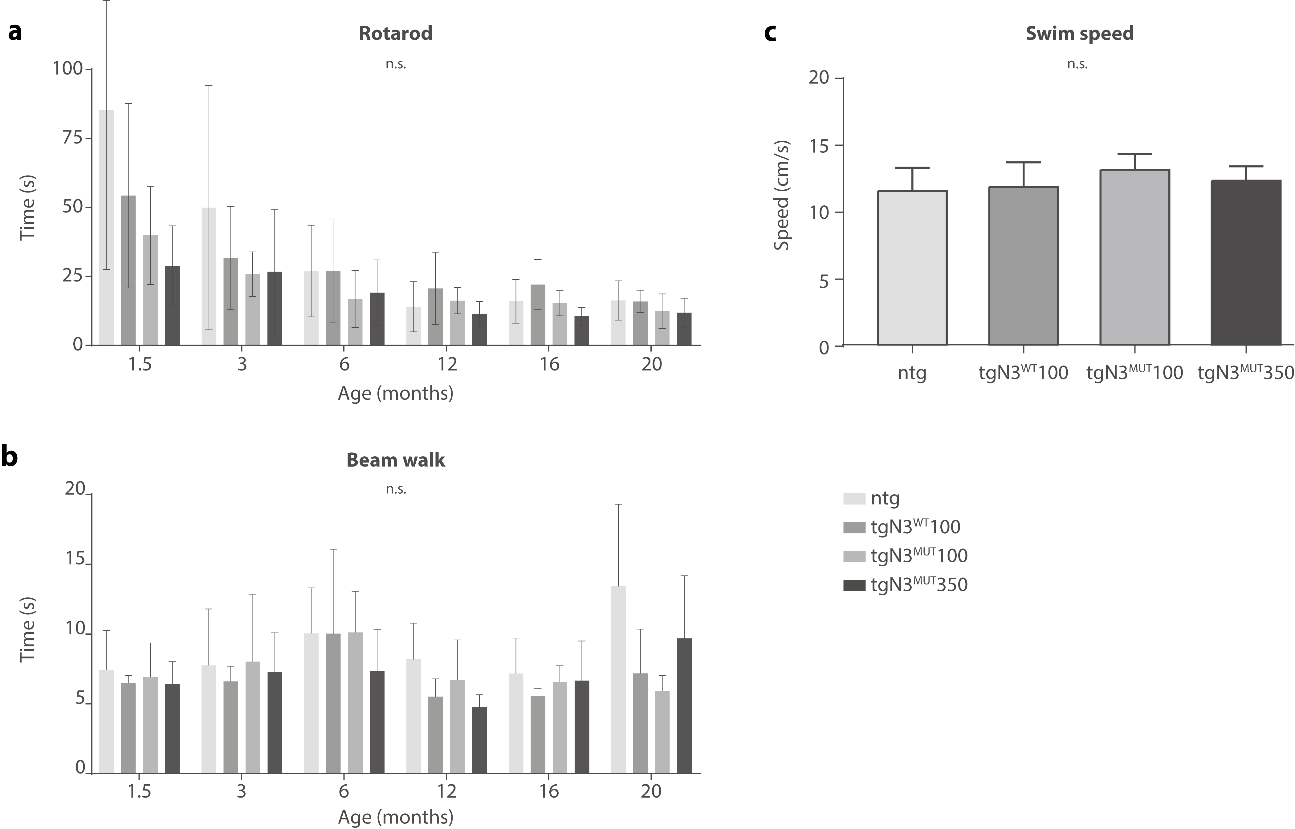
**

**Supplementary Data 6: CADASIL mice do not show motor dysfunction**

Bar charts showing (A) the time to complete a beam walk and (B) the time without falling of a rotarod for all groups (ntg, tgN3^WT^100, tgN3^MUT^100, tgN3^MUT^350) at various timepoints. No differences were seen between groups on the beam walk and rotarod motor function tests. (C) Average swimming speed from all trainings and reversal trainings was similar between groups. Graphs represent mean±SD; n.s. = non significant.

**Supplementary Data 7: Physiology parameters during cerebral hemodynamic assessment at baseline and at CO_2_ challenge in CADASIL mice**

|  | Baseline mean (sd)  *P-value* ^a^ | CO_2_ challenge  mean (sd)  *P-value* ^a^ | *P*-value ^b^ |
| --- | --- | --- | --- |
|  |  |  |  |
| **Body temperature (ºC)** | n.s. | n.s. |  |
| ntg | 35.4 (2.0) | 35.3 (2.4) | n.s. |
| tgN3^WT^100 | 35.3 (0.4) | 35.6 (0.5) | n.s. |
| tgN3^MUT^100 | 34.4 (0.5) | 34.5 (0.9) | n.s. |
| tgN3^MUT^350 | 35.2 (1.3) | 35.5 (1.2) | *P*=0.006 |
|  |  |  |  |
| **Respiration (/min)** | n.s. | n.s. |  |
| ntg | 154 (34) | 157 (33) | n.s. |
| tgN3^WT^100 | 125 (32) | 127 (45) | n.s. |
| tgN3^MUT^100 | 112 (45) | 131 (37) | n.s. |
| tgN3^MUT^350 | 160 (28) | 191 (50) | *P*=0.031 |
|  |  |  |  |
| **Oxygenation (%)** | n.s. | n.s. |  |
| ntg | 78 (17) | 77 (16) | n.s. |
| tgN3^WT^100 | 72 (15) | 72 (18) | n.s. |
| tgN3^MUT^100 | 74 (15) | 85 (4) | n.s. |
| tgN3^MUT^350 | 86 (11) | 90 (7) | n.s. |
|  |  |  |  |
| **Heart rate (/min)** | n.s. | *P*=0.009 ^c^ |  |
| ntg | 306 (73) | 315 (82) ^c^ | n.s. |
| tgN3^WT^100 | 225 (20) | 209 (22) ^c^ | n.s. |
| tgN3^MUT^100 | 300 (115) | 269 (23) | n.s. |
| tgN3^MUT^350 | 285 (51) | 290 (30) | n.s. |

^a^ *P*-value per tested physiology parameter represent ANOVA *P*-value for testing differences between groups at baseline and differences between groups at challenge.

^b^ *P*-value represent paired student’s *t*-test between baseline and challenge values of the parameter within the respective mouse strain.

^c^ Heart rate during CO_2_ challenge was significantly different between the different mouse strains. Post-hoc testing showed that tgN3^WT^100 mice had significantly lower heart rates than ntg (*P*=0.006).
n.s. = non significant.

**
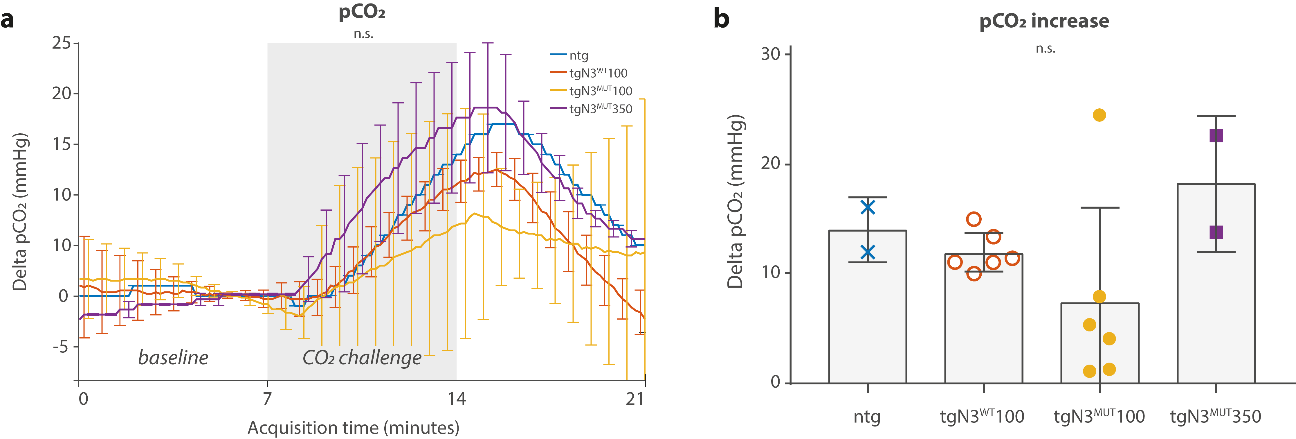
**

**Supplementary Data 8: pCO_2_ in blood increase upon CO­_2_ challenge
(A)** Absolute pCO_2_ increase is plotted over time. pCO_2_ levels were normalized to zero at baseline. **(B)** No significant differences in pCO_2_ increase between the mouse strains (ntg, tgN3^WT^100, tgN3^MUT^100, tgN3^MUT^350). Please note that pCO_2_ measurements of only a subset of mice is plotted (ntg n=1; tgN3^WT^100 n=6; tgN3^MUT^100 n=6; tgN3^MUT^350 n=2) as the pCO_2_ datasets of the other mice were lost due to technical reasons. Graphs represent mean±SD; n.s. = non significant.

**
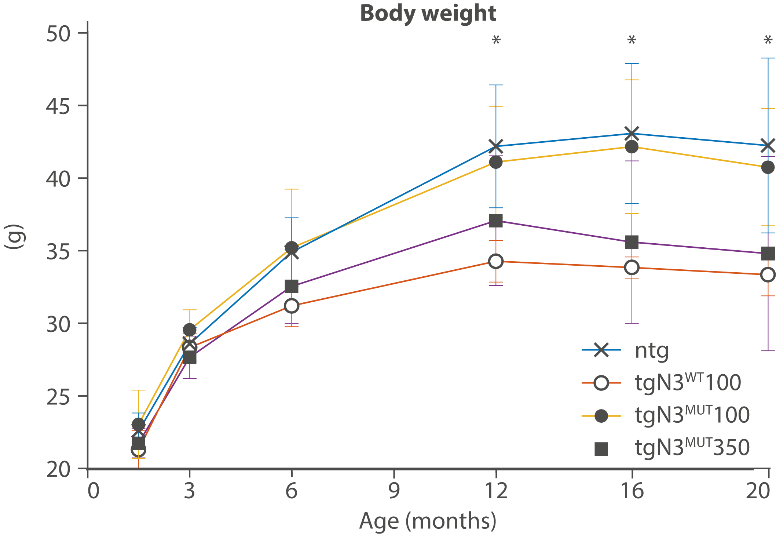
**

**Supplementary Data 9: Reduced body weight in tgN3^MUT^350 and tgN3^WT^100 mice compared to ntg and tgN3^MUT^100 mice**

Mice showed similar weights at younger ages, but from the age of 12 months, tgN3^WT^100 mice were significantly lighter than ntg mice, and from the age of 16 months, tgN3^WT^100 and tgN3^MUT^350 mice were significantly lighter than tgN3^MUT^100 and ntg mice (ntg 43.1±4.8 g, tgN3^WT^100 33.8±0.7 g, tgN3^MUT^100 42.2±4.6 g, tgN3^MUT^350 35.6±5.6 g, *P*=0.003; ANOVA). **P*<0.05. Graph represents mean±SD.
