# Supplementary material for: Progression and Classification of Granular Osmiophilic Material (GOM) Deposits in Functionally Characterized Human NOTCH3 Transgenic Mice
Source: Transl Stroke Res. 2019 Oct 30;11(3):517–27. doi: 10.1007/s12975-019-00742-7 (PMC7235067; doi:10.1007/s12975-019-00742-7)
Supplement: Supplementary file 2 — (DOCX 94 kb) [file 12975_2019_742_MOESM2_ESM.docx]

# Supplementary Methods

**Progression and classification of granular osmiophilic material (GOM) deposits in functionally characterized human NOTCH3 transgenic mice**

*Running head: GOM stages in CADASIL*

*Translational Stroke Research*

Gido Gravesteijn^1^, Leon P. Munting^2^, Maurice Overzier^3^, Aat A. Mulder^4^, Ingrid Hegeman^5^, Marc Derieppe^2,6^, Abraham J. Koster^4^, Sjoerd G. van Duinen^5^, Onno C. Meijer^7^, Annemieke Aartsma-Rus^3^, Louise van der Weerd^2,3^, Carolina R. Jost^4^_,_ Arn M.J.M. van den Maagdenberg^3,8^, Julie W. Rutten^1,3*^, Saskia A.J. Lesnik Oberstein^1*§^

^1^ Department of Clinical Genetics, Leiden University Medical Center, Albinusdreef 2, 2300 RC Leiden, The Netherlands.
^2^ Department of Radiology, Leiden University Medical Center, Albinusdreef 2, 2300 RC Leiden, The Netherlands.
^3^ Department of Human Genetics, Leiden University Medical Center, Albinusdreef 2, 2300 RC Leiden, The Netherlands.
^4^ Department of Cell and Chemical Biology, Leiden University Medical Center, Albinusdreef 2, 2300 RC Leiden, The Netherlands.
^5^ Department of Pathology, Leiden University Medical Center, Albinusdreef 2, 2300 RC Leiden, The Netherlands.
^6^ Department of Pediatric Neuro-Oncology, Prinses Máxima Center for Pediatric Oncology, Heidelberglaan 25, 3584 CS, Utrecht, The Netherlands.
^7^ Department of Internal Medicine, Leiden University Medical Center, Albinusdreef 2, 2300 RC Leiden, The Netherlands.
^8^ Department of Neurology, Leiden University Medical Center, Albinusdreef 2, 2300 RC Leiden, The Netherlands.
* Shared last authorship.
^§^ Corresponding author: Saskia Lesnik Oberstein, [lesnik@lumc.nl](mailto:lesnik@lumc.nl).

## Content

Suppl. Methods 1: Neuroimaging and CVR assessment p.2
Suppl. Methods 2: Cognitive assessment (Morris Water Maze) p.6
Suppl. Methods 3: Motor function tests p.7
Suppl. Methods 4: Quantification of vacuolization in mouse brain p.7
Supplementary References p.7

## Supplementary Methods 1: Neuroimaging and CVR assessment

Anaesthesia and physiological monitoring during neuroimaging

Mice were anesthetized with 3.5% isoflurane in medical air enriched with oxygen (75% air, 25% oxygen) for 4 minutes, followed by a continuous flow of 2% isoflurane during positioning of the mouse in the animal bed of the MRI scanner. A subcutaneous catheter was placed to infuse medetomidine (Dexdomitor, Vetoquinol SA, Lure, France [a solution without the inactive enantionmer levomedetomidine]) with a syringe pump (Univentor 802, Univentor High Precision Instruments, Zejtun, Malta). At the beginning of image acquisition, a bolus of 0.15 mg/kg medetomidine was injected, and 10 minutes later followed by a continuous infusion of 0.30 mg/kg/hr medetomidine. During those 10 minutes, the isoflurane concentration was slowly reduced to 0%, while the administration of the air/oxygen mixture was continued. During image acquisition, respiration was registered using a pressure pad placed underneath the animal. Heart rate and pulse oxygenation (both SA instruments, New York, USA) were recorded using an infrared probe around the leg. Changes in transcutaneous (tc)-pCO_2_ values were measured using a tc-pCO_2_ sensor (TCM radiometer, Zoetermeer, The Netherlands) placed on shaved skin on the flank of the animal. Temperature was maintained using a feedback-controlled waterbed (Medres, Cologne, Germany). Directly after neuroimaging, still under anaesthesia, mice were decapitated and tissue was collected.

MR image acquisition for cerebral hemodynamic assessment

MR images were acquired with a 7 T Bruker PharmaScan (Ettlingen, Germany) using a 23 mm volume coil. Anatomical T2 Turbo RARE scans were acquired in all three directions for planning of the arterial spin labelling (ASL) scans with the following parameters: TE/TR = 35.0 ms/2,500 ms, 0.7 mm slice thickness without a slice gap, 1 average, matrix of 256 by 256, field of view of 21.55 by 21.55 mm, RARE factor of 8, and a bandwidth of 36.7 kHz. To aid image registration, three slices of 1.5 mm thick with the same imaging parameters as above and the same geometry as the pseudo-continuous arterial spin labeling (pCASL) scans were acquired. The pCASL sequence was used to image the cerebral blood flow (CBF) and cerebrovascular reactivity (CVR, i.e. relative CBF increase). The label and control interpulse-phases of the sequence were optimized beforehand, to correct for off-resonance effects [1]. The labelling plane was placed in the neck, 1.0 cm from isocenter. Three 1.5 mm thick single-shot, spin-echo - Echo Planar Imaging (EPI) slices were used with a slice gap of 1 mm, where the middle slice was placed at isocenter and at -0.75 mm Bregma. The following imaging parameters were used: TE/TR = 16.8 ms/3,520 ms, labelling duration (τ) of 3,000 ms, post-label delay (PLD) of 300 ms, FOV of 21.55 x 21.55 mm and a matrix of 96 x 96. 180 pairs of label/control images (repetitions) were acquired, with a total pCASL imaging duration of 21 minutes. During the challenge, i.e. between minute 7 and 14 and between repetition 60 to repetition 120, 7.5% CO_2_ was added to the gas mixture. T1 maps were acquired using an inversion recovery EPI with 18 inversion times and with the same geometry as the pCASL sequence. Labelling efficiency (α) was measured 0.3 cm downstream the labelling plane with a flow-compensated, ASL-encoded FLASH sequence. The latter two sequences were used to support CBF-quantification. After the pCASL scan, three additional anatomical scans were acquired with the following details: (1) a high resolution T2W sequence with TE/TR = 39 ms/2,200 ms, a matrix of 384 x 384, 9 slices with a thickness of 0.7 mm, a 0.3 mm slice gap and 8 averages; (2) a T2W-Fluid-Attenuated Inversion Recovery (FLAIR) sequence with TE/TR = 37 ms/1,000 ms, a matrix of 192 x 192, 9 slices with a thickness of 1.0 mm, without a slice gap and 3 averages; and (3) a Susceptibility-Weighted Imaging (SWI) sequence with TE/TR = 18 ms/350 ms, a matrix of 384 x 384, 9 slices with a thickness of 0.7 mm, a 0.3 mm slice gap and 5 averages. These three sequences were acquired in the same orientation and with the same FOV as the pCASL scan.

MR image registration and processing

Individual pCASL EPIs and individual inversion recovery EPIs were aligned to the first label magnitude image of the pCASL sequence using a MATLAB monomodal rigid body registration with 300 iterations (MATLAB version R2016a, Mathworks, Natick, USA). The anatomical T2RARE images of one dataset were used to delineate the cortex and the full brain. These regions of interest (ROIs) were automatically propagated to the T2RARE images of the other datasets, and subsequently to the EPIs of every dataset. An edge-based variational method for non-rigid multimodal registration was used to propagate the ROIs [2]. The results of both propagation steps were verified for each dataset by an operator (L.P.M.).

Buxton’s general kinetic perfusion model was used to quantify cerebral blood flow (CBF) from the measured difference between label and control acquisition (Buxton et al., *Magn Reson Med*. 1998;40:383-396), *i.e.* the following equation was used:

$$CBF=\frac{\lambda\cdot\Delta M\cdot exp\left( \mathrm{PLD}/{T_{1b}} \right)}{2\cdot{\alpha\cdot T}_{1t}\cdot M_{0t}\cdot\left( 1-exp\left( -\tau/{T_{1t}} \right) \right)} (1)$$

Here, ΔM is the measured difference between the label and control EPIs, T1b is the T1 of blood at 7 T, assumed to be 2230 ms [3]. Also, it is assumed that at thermal equilibrium, the magnetization of arterial blood (M0b) may be approximated by M0t/λ, where M0t is the magnetization of tissue and λ is 0.9, the blood–brain partition coefficient of water [4]. Baseline CBF was defined as the mean CBF of the 20 repetitions before the onset of the CO_2_ challenge, which is equal to 2 minute 20 seconds before the onset. CBF during CO_2_ was defined as the mean of the 20 repetitions before the end of the CO_2_. These two estimates were used to calculate cerebrovascular reactivity (CVR) by using the following equation:

$$\mathrm{CVR}\left( \% \right)=100\cdot\frac{mean CBF during challenge-mean CBF during baseline}{mean CBF during baseline} (2)$$

MR image acquisition for blood brain barrier assessment using Gadolinium

Blood brain barrier function was assessed in a second cohort of ntg (n=4), tgN3WT100 (n=4), and tgN3MUT350 (n=5) mice at the age of 12 months by determining Gadolinium-induced signal enhancement on brain MRI after injection of Gadolinium. Gadolinium-DOTA (Dotarem, Guerbet, Cedex, France) was administered intra-peritoneally at 10 mmol/kg. MRI was performed using a series of consecutive T1W images with a RARE sequence (TR/TE = 870/11.7 ms, matrix size = 256 x 256, field-of-view = 20 x 20 mm^2^, number of averages = 6, slice thickness = 0.5 mm, coronal slices, RARE factor = 2), both before (1 image) and after (6 images) contrast agent administration. The 6 post-contrast images have been acquired immediately after the contrast agent injection, thus started at 0 min, 12 min, 24 min, 36 min, 48 min, and 60 min post-injection, respectively. Blood brain barrier leakage was calculated as percentage signal enhancement after contrast in a region-of-interest placed on the cortex at Bregma -2 mm. Signal enhancement was calculated as the relative change in signal intensity (SI) between pre- and the last post-contrast T1W image (ΔSI) as per the following equation, ΔSI= [ SI(t) – SI(0) ] / SI(0), where SI(t) is the signal intensity at time t after Gadolinium-DOTA injection and SI(0) is the signal intensity of the selected region-of-interest in the scan prior to Gadolinium-DOTA injection.

## Supplementary Methods 2: Cognitive assessment

A Morris water maze protocol was used to assess cognitive function, as it is considered to be a valid measure of hippocampal dependent spatial navigation and reference memory. The swimming pool (diameter 138cm) was filled with opaque white water (by adding non-toxic paint) which was maintained at a temperature of 20-22°C. A platform (diameter 10.8 cm, platform pool ratio 1:163) was placed 5-8 mm under the water surface in the north-west quadrant and south-east quadrant during the training phase and reversal training phase, respectively. Ten cm from the pool, three high contrast figures were placed to facilitate spatial navigation. To prevent praxic and taxic navigation rather than spatial navigation, mice were released from different locations during the experiment.

Three days before starting the training phase, mice were allowed to swim in the pool for 2 min. Mice underwent 4 trials for 5 or 4 days during the training phase or reversal training phase, respectively, where they had a maximum of 120 seconds per trial to locate the hidden platform. After the trail, regardless whether they found the platform, mice were placed on the platform for 15 seconds allowing them to remember the location. Three days after the training phase and reversal training phase, mice were placed for 2 minutes in the swimming pool of which the platform was removed (no platform test and reversal no platform test, respectively). Mice were recorded using the Noldus EthoVision® XT Base software (version 11.5). Path length to platform was used as outcome measure for (reversal) training phase and time per quadrant for the no-platform tests.
**
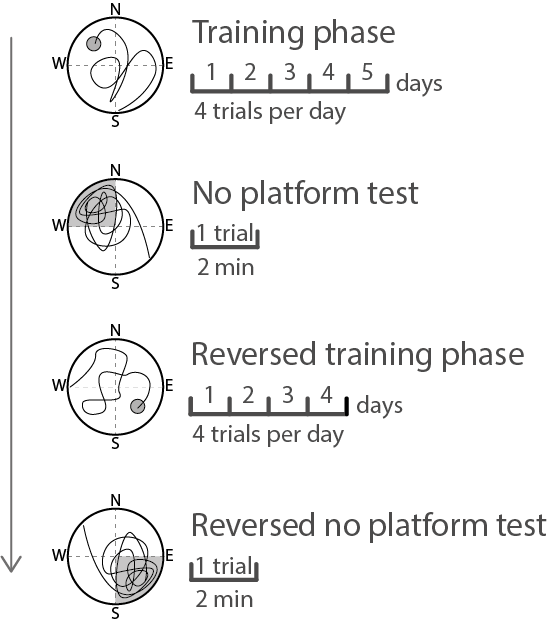
**

**Figure: Graphical summary experimental design Morris water maze protocol**

## Supplementary Methods 3: Motor function tests

For rotarod tests, mice had to run on a rotating rod (speed gradually increasing up to 45 rpm) for a maximum of 5 minutes. For beam walk tests, time to cross a beam of 80 cm with a diameter of 11 mm was recorded. As we noted that all mice had difficulty performing the task, from the age of 6 months, the diameter of the beam was increased to 17 mm. Mice took three trials per time point and the average time was analysed for both tests. Average swimming speed was calculated based on all MWM trials.

## Supplementary Methods 4: Quantification of vacuolization in mouse brain

Two 5-µm brain sections per mouse were analysed after staining them with Klüver-Barrera luxol fast blue staining according to standard protocol. Corpus callosum was captured at 400x with the same capture settings for all sections. Regions-of-interest were manually draws around the corpus callosum, thereby excluding blood vessel area if vessels were present in the image. Vacuoles were quantified using ImageJ thresholding for the vacuole areas. Vacuole area is expressed as vacuole area percentage compared to total region-of-interest area. Multiple measurements were averaged per mouse.

## Supplementary References

1. Hirschler L, Debacker CS, Voiron J, Köhler S, Warnking JM, Barbier EL. Interpulse phase corrections for unbalanced pseudo-continuous arterial spin labeling at high magnetic field. Magn Reson Med. 2018;79:1314–24.

2. Denis De Senneville B, Zachiu C, Ries M, Moonen C. EVolution: An edge-based variational method for non-rigid multi-modal image registration. Phys Med Biol. IOP Publishing; 2016;61:7377–96.

3. Dobre MC, Uğurbil K, Marjanska M. Determination of blood longitudinal relaxation time (T1) at high magnetic field strengths. Magn Reson Imaging [Internet]. 2007;25:733–5. Available from: http://www.ncbi.nlm.nih.gov/pubmed/17540286

4. Herscovitch P, Raichle ME. What is the correct value for the brain--blood partition coefficient for water? J Cereb Blood Flow Metab [Internet]. 1985;5:65–9. Available from: http://www.ncbi.nlm.nih.gov/pubmed/3871783
